# Supplementary material for: Exact exchange-correlation potentials from ground-state electron densities
Source: Nat Commun. 2019 Oct 3;10:4497. doi: 10.1038/s41467-019-12467-0 (PMC6776552; doi:10.1038/s41467-019-12467-0)
Supplement: Supplementary file 1 — Supplementary Information [file 41467_2019_12467_MOESM1_ESM.pdf]

Supplementary Information

Exact exchange-correlation potentials from ground-state electron densities

Kanungo et al.

Supplementary Note 1 — Finite-element basis and Cusp Condition

In the finite element method, the spatial domain of interest is discretized into subdomains called finite elements. The finite element basis is constructed from piecewise polynomial functions that have a compact support on the finite elements, thus rendering locality to these basis functions. Typically, the polynomials are constructed using Lagrange interpolating functions. For example, in the 1D case, given a finite-element e with a set of nodes $\{x_1, x_2, \ldots, x_{p+1}\}$ (not necessarily equi-spaced), we can construct $p+1$ polynomials, each of order p, given by

$$N_i^{e,p}(x) = \prod_{\substack{j=1 \\ j \neq i}}^{p+1} \frac{x - x_j}{x_i - x_j} \,. \tag{1}$$

For the 3D case, the basis is constructed by taking tensor products of the 1D Lagrange polynomials. We remark that, as per the above construction, the finite-element basis has a C^0 continuity at the element boundary. We illustrate this through a 1D cubic finite-element basis, as shown in Supplementary Figure 1. In this figure, the black circles mark the C^0 continuity at the element boundary. This C^0 continuity of the basis at the element boundaries is vital to ensuring the cusp in $\rho(\mathbf{r})$ at the nuclei. To elaborate, in our calculations, we construct an adaptive finite-element mesh such that the nuclei are positioned on corner nodes of elements. Thus, the resultant finite-element basis, by design, admits cusps in the Kohn-Sham orbitals ($\psi_i(\mathbf{r})$) (and hence in $\rho(\mathbf{r})$) at the nuclear positions.

Supplementary Discussion

Verification against LDA based $\rho_{\mathbf{data}}$

We provide more details on the accuracy of our inverse DFT approach for the verification tests we conducted against LDA based ρ_{data}. Supplementary Figure 2 presents the comparison of the inverted v_{xc} against $v_{\text{xc}}^{\text{LDA}}[\rho_{\text{data}}]$, in both small and large r regimes. As evident from the plots, we obtain remarkable agreement between the two, in both near- and far-field. We also elaborate on the accuracy of the v_{xc} for the 1,3-dimethylbenzene molecule, obtained from the inverse DFT calculation with LDA based ρ_{data} (refer to Figure 3 in the main article) . Supplementary Figure 3 shows the relative error in v_{xc} (i.e., $\left|v_{\text{xc}} - v_{\text{xc}}^{\text{LDA}}[\rho_{\text{data}}\right| / \left|v_{\text{xc}}^{\text{LDA}}[\rho_{\text{data}}]\right|$). As evident from the figure, we attain good accuracy for the v_{xc}, devoid of any spurious oscillations, even for this relatively large system (by inverse DFT standards).

L^1 error in densities

As mentioned in the main article, we used the L^2 error in the density, given by $\|\rho(\mathbf{r}) - \rho_{\text{data}}(\mathbf{r})\|_{L^2} = \sqrt{\int \left(\rho(\mathbf{r}) - \rho_{\text{data}}(\mathbf{r})\right)^2 \, d\mathbf{r}}$, as the convergence criterion in our inverse calculations. This choice of L^2 error is motivated from the fact that the objective function in our PDE-constrained optimization (see Eq. 1 in the main article) is the square of the L^2 error in the density with $w(\mathbf{r}) = 1$. However, several prior works have reported the L^1 error in density, given by $\|\rho(\mathbf{r}) - \rho_{\text{data}}(\mathbf{r})\|_{L^1} = \int |\rho(\mathbf{r}) - \rho_{\text{data}}(\mathbf{r})| \, d\mathbf{r}$. Therefore, for the benefit of a fair assessment of our work against previous efforts, we tabulate the L^1 error normalized by the number of electrons (N_e), i.e., $\|\rho(\mathbf{r}) - \rho_{\text{data}}(\mathbf{r})\|_{L^1} / N_e$. The normalization is introduced as the systems considered in this study span a wide range—from $N_e = 2$ to $N_e = 58$. Supplementary Table 1 lists the normalized L^1 error in density for the verification tests that we have performed using LDA based ρ_{data}. As evident, we obtain excellent accuracy for all the systems, including the 1,3-dimethylbenzene (C_8H_{10})—a large system by inverse DFT standards. Supplementary Table 2 lists the normalized L^1 error in density for all benchmark systems where the ρ_{data} was obtained from CI calculations. As evident from the table, we obtain excellent accuracy even in terms of the L^1 error for the range of systems considered. We remark that the error for the dissociated hydrogen molecule ($H_2(d)$) is an order of magnitude higher than other systems, largely attributed to the presence of longer-ranged correlations, which in turn, in the absence of accurate far-field densities, creates larger error while making use of approximate

boundary condition on v_{xc} (see the main article for a detailed explanation).

Rate of convergence

The rate of convergence, and the factors affecting it, are now discussed for the benchmark systems considered in this work. The most critical factor determining the rate of convergence of the inverse DFT algorithm is the Kohn-Sham HOMO-LUMO gap. To elaborate, for all systems with an appreciable HOMO-LUMO gap (i.e., all systems except $H_2(d)$), convergence was attained in 300-500 BFGS iterations. However, for $H_2(d)$, wherein the HOMO-LUMO gap is ~ 3 mHa, the algorithm took ~ 2500 BFGS iterations to attain convergence. Interestingly, our studies suggest that the size of the system is less critical to the rate of convergence. For instance, both H_2O and benzyne molecule took $\sim 2\times$ the number of iterations taken by $H_2(eq)$. Finally, the rate of convergence was only weakly dependent on the choice of initial guess. In our studies, two different initial guesses were used, starting with either the PW92 (LDA) potential or the LB94 (GGA) potential. While the final result is independent of the initial guess, the LB94 guess took $\sim 50 - 100$ lesser BFGS iterations to converge compared to the PW92 guess.

Comparison with existing methods

The proposed approach to the inverse DFT problem is now compared to prior approaches in terms of accuracy, robustness and computational viability, using results from existing literature. Given the importance of the inverse DFT problem, several attempts have been made at solving this over the past two decades. Broadly speaking, the approaches proposed can be classified into three major categories—(a) iterative update based algorithms [1, 2, 3, 4, 5, 6]; (b) constrained optimization approaches [7, 8, 9, 10, 11, 12]; (c) linear response based approaches [13]. Irrespective of the underlying approach, the two major factors that determine the accuracy of obtained $v_{\text{xc}}(\mathbf{r})$ are—(a) the completeness of the basis in which the Kohn-Sham orbitals ($\psi_i(\mathbf{r})$) and the $v_{\text{xc}}(\mathbf{r})$ are discretized, and (b) the accuracy of the input density $\rho_{\text{data}}(\mathbf{r})$, including the correct near-field (cusp at the nuclear positions) and far-field asymptotics. The major criteria for judging the accuracy of the resulting $v_{\text{xc}}(\mathbf{r})$ are the L^1 and L^2 errors in the KS density (i.e. the output density) that is produced by this $v_{\text{xc}}(\mathbf{r})$, as well as errors in the $v_{\text{xc}}(\mathbf{r})$ potential itself. These metrics will form the basis for our discussion that follows.

To begin with, we discuss the importance of using a complete basis to discretize the inverse DFT problem, which in this work is achieved by using a systematically convergent finite-element basis. To this end, we compare the results of our verification studies using ρ_{data}^{LDA} with similar results from published literature. This verification test allows for a direct assessment of the accuracy of the v_{xc} obtained using inversion, by comparing it against $v_{\text{xc}}^{\text{LDA}}[\rho_{\text{data}}]$. As noted in the main article, several attempts at this verification test have suffered from either non-unique solutions or had resulted in unphysical oscillations in v_{xc}, owing to the incompleteness of the Gaussian basis employed in these works. In particular, as demonstrated in [10, 11], the use of Gaussian basis results in errors in $\left|v_{\text{xc}}(\mathbf{r}) - v_{\text{xc}}^{\text{LDA}}[\rho_{\text{data}}]\right|$ in the range of $\mathcal{O}(10^0 - 10^1)$ (cf. Figure 2 in [10] and Figure 2 in [11]). A workaround to suppress this incomplete basis induced oscillation is to either introduce a regularization constraint on the v_{xc}, or to construct a balanced potential basis (for the corresponding orbital basis). Although these techniques alleviate the wild oscillations, the resulting v_{xc} still exhibits $\mathcal{O}(10^0)$ error (cf. Figures 4 and 6 in [10]; Figures 6 and 7 in [11]), and is sensitive to the choice of regularization parameters/balanced basis. On the other hand, we demonstrate that the use of finite-element basis, owing to its completeness, results in smooth v_{xc} with a tight accuracy of $\mathcal{O}(10^{-2})$, for similar LDA-based verification studies (refer Figure 2 in the main article, Supplementary Figures 2 and 3). A recent effort [12] employs additional constraints to obtain the most optimal v_{xc} in a given Slater basis. Although this approach results in better v_{xc} for similar verification studies, the normalized error in the density (i.e., $\left\|\rho(\mathbf{r}) - \rho_{\text{data}}(\mathbf{r})\right\|_{L^1}/N_e$) remains high ($\sim 3\times10^{-3} - 6\times10^{-4}$). In comparison, we obtain $\left\|\rho(\mathbf{r}) - \rho_{\text{data}}(\mathbf{r})\right\|_{L^1}/N_e$ in the range of $6\times10^{-6} - 8\times10^{-6}$ (cf. Supplementary Table 1), for similar verification studies. We remark that, in addition to these quantitative errors, the use of Gaussian basis has also resulted in qualitatively incorrect v_{xc}'s. For example, in [9], the v_{xc} for H_2, corresponding to CCSD based ρ_{data}, does not have a local maximum between the two H atoms, a feature present in both the exact as well approximate v_{xc}'s (such as LDA, GGA)—cf. Figure 5 in the main article. Thus, the above comparisons show that the finite-element basis simultaneously provides accurate potentials and densities in solving the inverse DFT problem.

Using accurate densities (ρ_{data}) is also important in the inverse DFT problem, and the presence of cusp in ρ_{data} at nuclei is particularly important. The vitality of the nuclear cusp has been highlighted in Figure 4 of the main article, wherein the lack of cusp in the Gaussian basis-set density induces wild oscillations in the v_{xc} near the nuclei. Similar Gaussian-density induced oscillations have also been observed in [14, 13, 5]. Thus, it is desirable to use ρ_{data} generated using Slater or other complete basis sets. However, the

difficulty of performing CI calculations in Slater basis (owing to the large computational cost involved in the four-center integrals associated with Slater basis) restricts the availability of physically meaningful densities to atomic and small molecular systems. To this end, we provide a practically useful solution by adding a small $\Delta\rho$ correction to ρ_{data} (cf. Eq. 10 in the main article), so as to correct for the missing cusp at the nuclei. This has enabled us to obtain exact v_{xc}'s of remarkable quality using Gaussian densities, with an accuracy of $\mathcal{O}(10^{-5}-10^{-4})$ in $\|\rho(\mathbf{r})-\rho_{\text{data}}(\mathbf{r})\|_{L^1}/N_e$ (cf. Supplementary Table 2), for systems comprising up to 40 electrons. Similar accuracy has been obtained in 1D (atomic) systems [2, 13, 4, 6], wherein Slater densities (or densities obtained on a radial grid) are used in conjunction with solution of 1D (radial) Kohn-Sham equations via numerical integration. An extension of these techniques to 3D systems remains computationally challenging, due to the difficulty in obtaining accurate Slater densities for large molecular systems as well as adapting the numerical approach used in these works for the solution of Kohn-Sham equations to 3D setting. To this end, the use of finite-elements, as employed in this work, affords both the availability of accurate densities and the computational viability to handle large 3D molecular systems.

In conclusion, the proposed approach—that combines (i) PDE-constrained optimization; (ii) the use of finite-elements that can be systematically converged to completeness; (iii) the cusp-correction to Gaussian densities; (iv) the use of far-field boundary conditions that ensures the correct asymptotic behavior for v_{xc}—provides a solution to the inverse DFT problem that is more accurate (and often more stable) than prior attempts. This allows our approach to competently handle large molecular systems, including systems that are strongly correlated.

Supplementary Figures

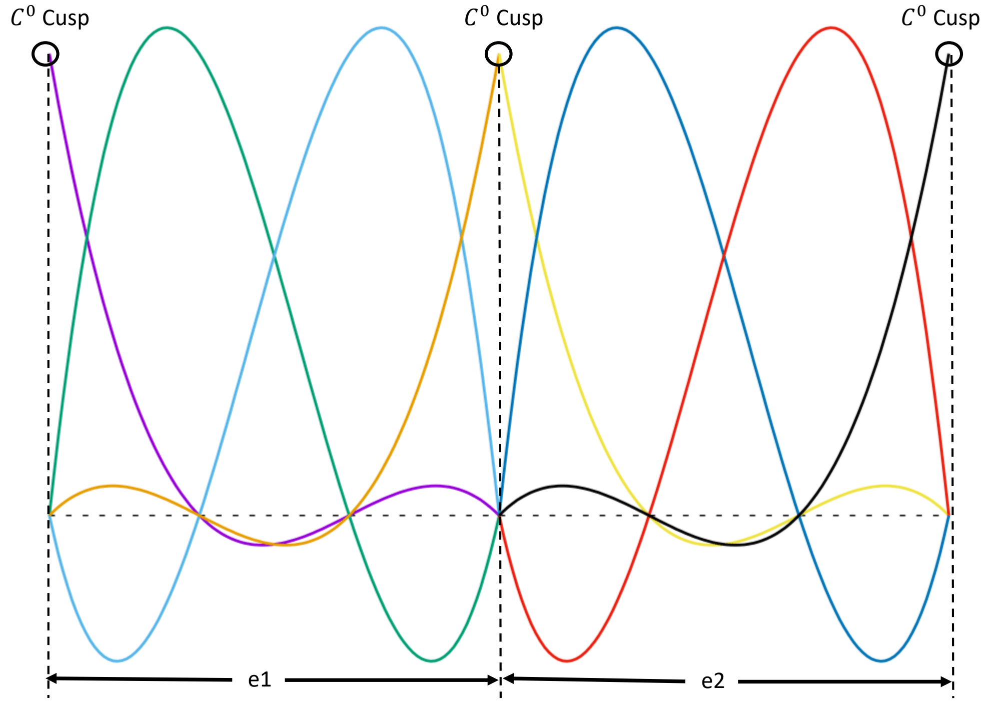

Supplementary Figure 1: Illustration of two adjacent 1D cubic finite-elements—e1 and e2. The vertical dashed lines denote the boundary between adjacent elements. The black circles highlight the C^0 continuity (cusp) of the basis at the element boundary.

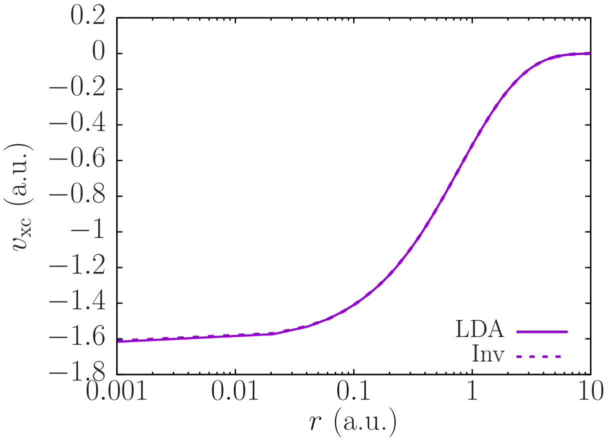

(a)

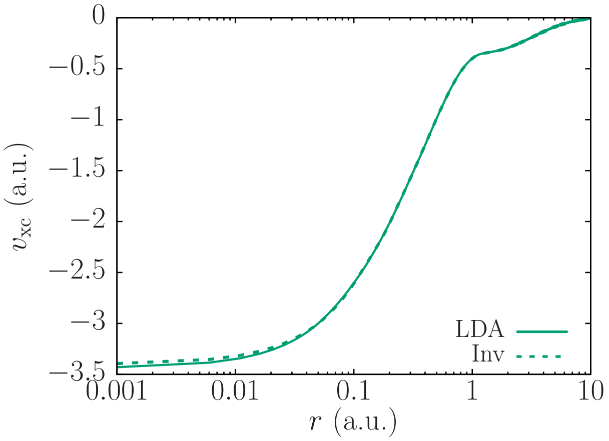

(b)

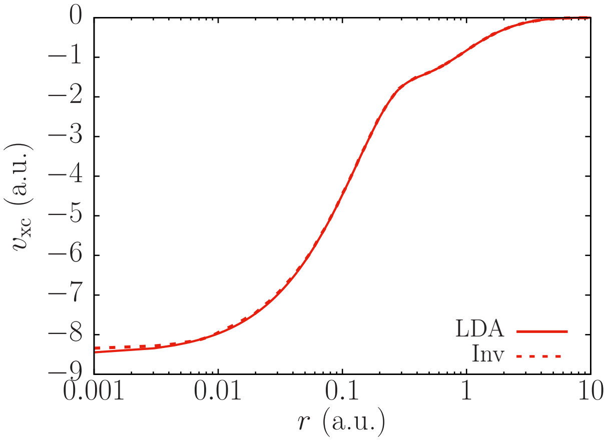

(c)

Supplementary Figure 2: The exchange-correlation potential (v_{xc}) for various atomic systems, each corresponding to the local density approximation (LDA) based density (ρ_{data}). The dashed line corresponds to the exchange-correlation potential obtained from the inverse DFT calculation, and the solid line corresponds to the LDA exchange-correlation potential. The atomic systems considered are: **(a)** He; **(b)** Be; **(c)** Ne.

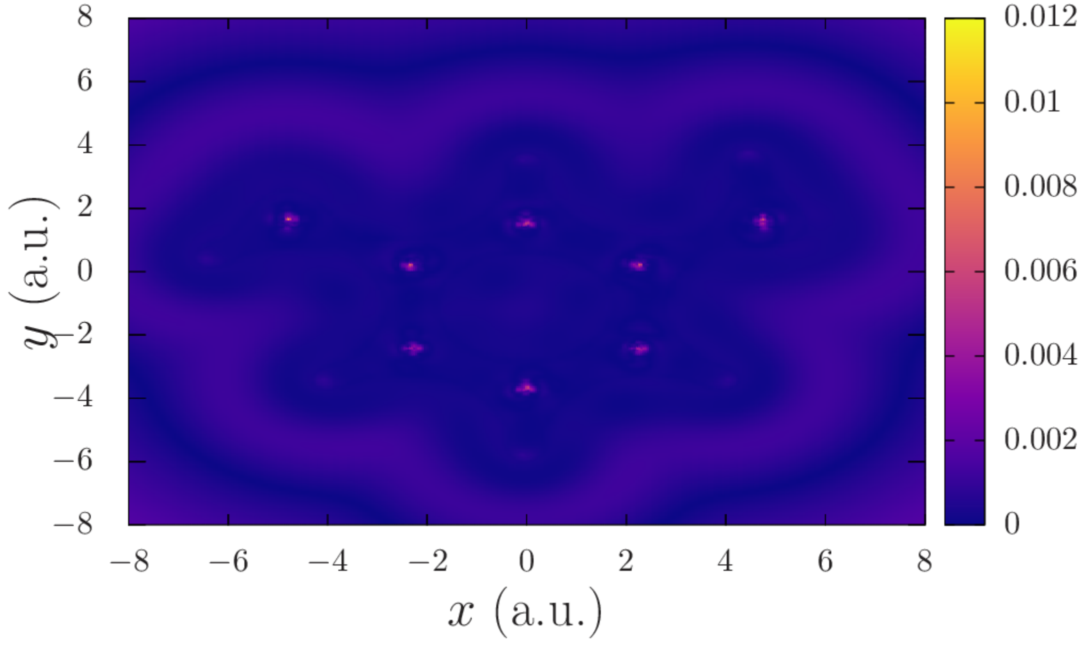

Supplementary Figure 3: Relative error in the v_{xc} for 1,3-dimethylbenzene (C_8H_{10}) obtained from the inverse DFT calculation with the local density approximation (LDA) based ρ_{data}, presented on the plane of the benzene ring.

Supplementary Tables

Supplementary Table 1: $\|\rho(\mathbf{r}) - \rho_{\text{data}}(\mathbf{r})\|_{L^1} / N_e$ for the verification tests against the local density approximation (LDA) based ρ_{data}.

He	Be	Ne	C_8H_{10}
8.1×10^{-6}	8.4×10^{-6}	7.2×10^{-6}	6.8×10^{-6}

Supplementary Table 2: $\|\rho(\mathbf{r}) - \rho_{\text{data}}(\mathbf{r})\|_{L^1} / N_e$ for ρ_{data} obtained from CI calculations.

$H_2(eq)$	$H_2(2eq)$	$H_2(d)$	H_2O	C_6H_4
3.5×10^{-5}	4.7×10^{-5}	4.2×10^{-4}	3.4×10^{-5}	8.2×10^{-5}

Supplementary Table 3: Coordinates for all benchmark systems (in a.u.).

$H_2(eq)$				
	H	−0.699	0.0	0.0
	H	0.699	0.0	0.0
$H_2(2eq)$				
	H	−1.415	0.0	0.0
	H	1.415	0.0	0.0
$H_2(d)$				
	H	−3.7795	0.0	0.0
	H	3.7795	0.0	0.0
H_2O				
	O	0.0	0.0	0.0
	H	0.0	1.8897	0.0
	H	1.8327	−0.4607	0.0
C_8H_{10}				
	C	2.2976	0.2359	0.0
	C	−0.00276	1.50778	0.0
	C	−2.3137	0.2284	0.0
	C	−2.284	−2.3996	0.0
	C	−0.00226	−3.7077	0.0
	C	2.2689	−2.4046	0.0
	C	4.7677	1.6546	0.0
	C	−4.7636	1.6818	0.0
	H	−0.00426	3.5568	0.0
	H	−4.0496	−3.4334	0.0
	H	−0.00443	−5.7529	0.0
	H	4.0371	−3.4356	0.0
	H	5.8985	1.1838	1.6606
	H	4.464	3.6909	0.0
	H	5.89852	1.1838	−1.6606
	H	−4.9107	2.8976	1.6608
	H	−6.3865	0.4148	0.0
	H	−4.9107	2.8976	−1.6608

Continued on next page

Supplementary Table 3: *Continuing from previous page*

C_6H_4				
	C	1.17276	−2.085	0.0
	C	−1.17276	−2.0850	0.0
	C	−2.7274	−0.0054	0.0
	C	−1.3202	2.2231	0.0
	C	1.3202	2.2231	0.0
	C	2.7274	−0.0054	0.0
	H	−4.7590	−0.00896	0.0
	H	−2.303	4.0071	0.0
	H	2.303	4.0071	0.0
	H	4.7590	−0.00896	0.0

Supplementary References

[1] Wang, Y. & Parr, R. G. Construction of exact Kohn-Sham orbitals from a given electron density. *Phys. Rev. A* **47**, R1591–R1593 (1993). URL https://link.aps.org/doi/10.1103/PhysRevA.47.R1591.

[2] van Leeuwen, R. & Baerends, E. J. Exchange-correlation potential with correct asymptotic behavior. *Phys. Rev. A* **49**, 2421–2431 (1994). URL https://link.aps.org/doi/10.1103/PhysRevA.49.2421.

[3] Gritsenko, O. V., van Leeuwen, R. & Baerends, E. J. Molecular Kohn-Sham exchange-correlation potential from the correlated ab initio electron density. *Phys. Rev. A* **52**, 1870–1874 (1995). URL https://link.aps.org/doi/10.1103/PhysRevA.52.1870.

[4] Peirs, K., Van Neck, D. & Waroquier, M. Algorithm to derive exact exchange-correlation potentials from correlated densities in atoms. *Phys. Rev. A* **67**, 012505 (2003). URL https://link.aps.org/doi/10.1103/PhysRevA.67.012505.

[5] Kadantsev, E. S. & Stott, M. J. Variational method for inverting the Kohn-Sham procedure. *Phys. Rev. A* **69**, 012502 (2004). URL https://link.aps.org/doi/10.1103/PhysRevA.69.012502.

[6] Ryabinkin, I. G. & Staroverov, V. N. Determination of Kohn-Sham effective potentials from electron densities using the differential virial theorem. *J. Chem. Phys.* **137**, 164113 (2012). URL https://doi.org/10.1063/1.4763481.

[7] Zhao, Q., Morrison, R. C. & Parr, R. G. From electron densities to Kohn-Sham kinetic energies, orbital energies, exchange-correlation potentials, and exchange-correlation energies. *Phys. Rev. A* **50**, 2138–2142 (1994). URL https://link.aps.org/doi/10.1103/PhysRevA.50.2138.

[8] Tozer, D. J., Ingamells, V. E. & Handy, N. C. Exchange-correlation potentials. *J. Chem. Phys.* **105**, 9200–9213 (1996). URL https://doi.org/10.1063/1.472753.

[9] Wu, Q. & Yang, W. A direct optimization method for calculating density functionals and exchange-correlation potentials from electron densities. *J. Chem. Phys.* **118**, 2498–2509 (2003). URL https://aip.scitation.org/doi/abs/10.1063/1.1535422.

[10] Heaton-Burgess, T., Bulat, F. A. & Yang, W. Optimized effective potentials in finite basis sets. *Phys. Rev. Lett.* **98**, 256401 (2007). URL https://link.aps.org/doi/10.1103/PhysRevLett.98.256401.

[11] Bulat, F. A., Heaton-Burgess, T., Cohen, A. J. & Yang, W. Optimized effective potentials from electron densities in finite basis sets. *J. Chem. Phys.* **127**, 174101 (2007). URL https://doi.org/10.1063/1.2800021.

[12] Jacob, C. R. Unambiguous optimization of effective potentials in finite basis sets. *J. Chem. Phys.* **135**, 244102 (2011). URL https://doi.org/10.1063/1.3670414.

[13] Schipper, P. R. T., Gritsenko, O. V. & Baerends, E. J. Kohn-Sham potentials corresponding to Slater and Gaussian basis set densities. *Theor. Chem. Acc.* **98**, 16–24 (1997). URL https://doi.org/10.1007/s002140050273.

[14] Mura, M. E., Knowles, P. J. & Reynolds, C. A. Accurate numerical determination of Kohn-Sham potentials from electronic densities: I. Two-electron systems. *J. Chem. Phys.* **106**, 9659–9667 (1997). URL https://doi.org/10.1063/1.473838.
